# Supplementary material for: African Swine Fever Virus and Host Response: Transcriptome Profiling of the Georgia 2007/1 Strain and Porcine Macrophages
Source: J Virol. 2022 Mar 9;96(5):e01939-21. doi: 10.1128/jvi.01939-21 (PMC8906413; doi:10.1128/jvi.01939-21)
Supplement: Supplemental file 8 — Fig. S1 and S2. Download jvi.01939-21-s0008.pdf, PDF file, 0.9 MB [file jvi.01939-21-s0008.pdf]

Supplementary Figures

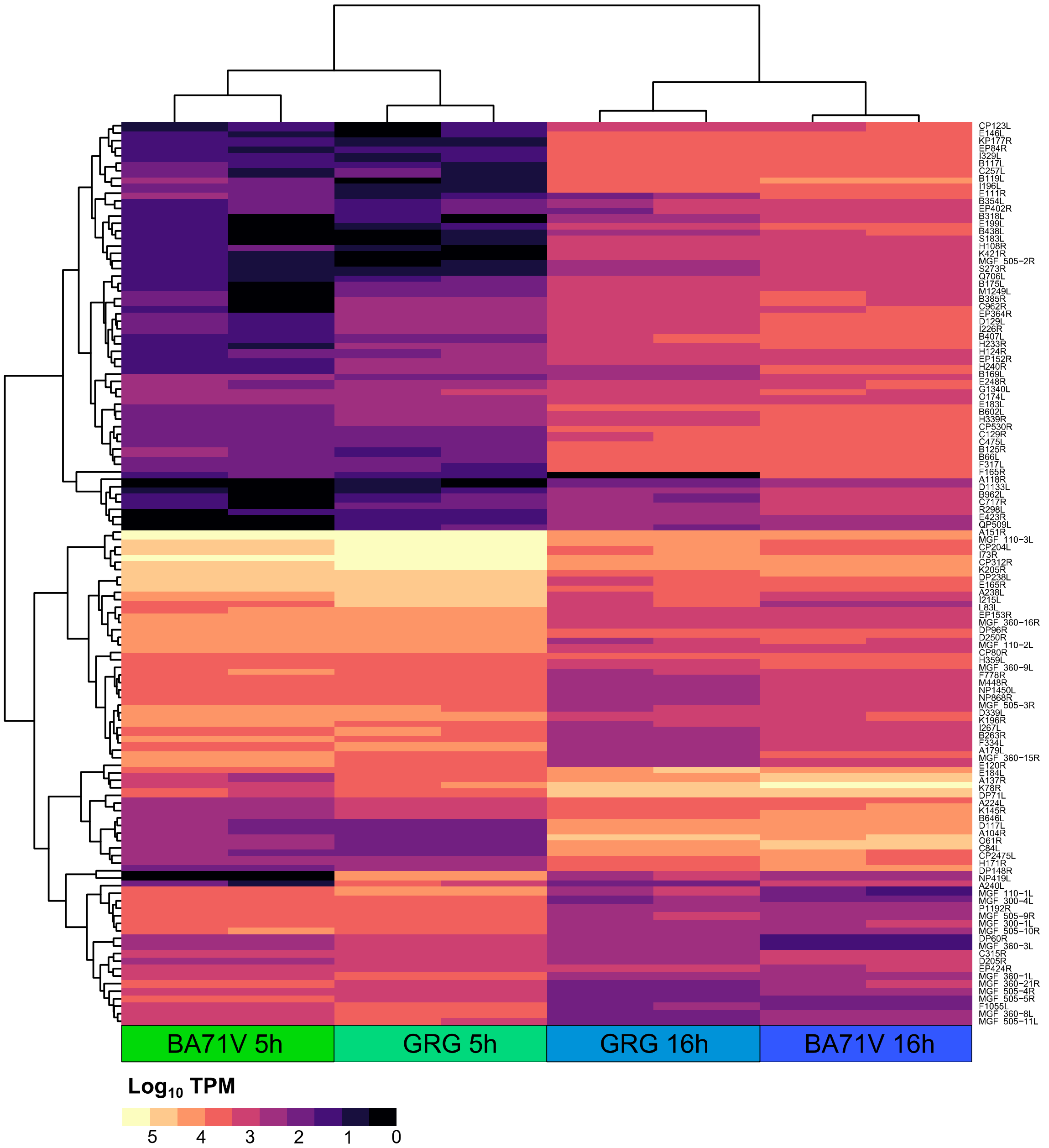

Supplementary Figure 1. Clustered heatmap of ASFV gene expression for the genes shared between BA71V and GRG that showed significant differential expression. R package ‘pheatmap’-generated clustered heatmap of per-gene RPM values for genes shared between GRG and BA71V, across time-points (5 hpi and 16 hpi), strains, with biological replicates as separate columns. Gene names for each row are listed on the right and both rows and columns were clustered according to Euclidean distance.

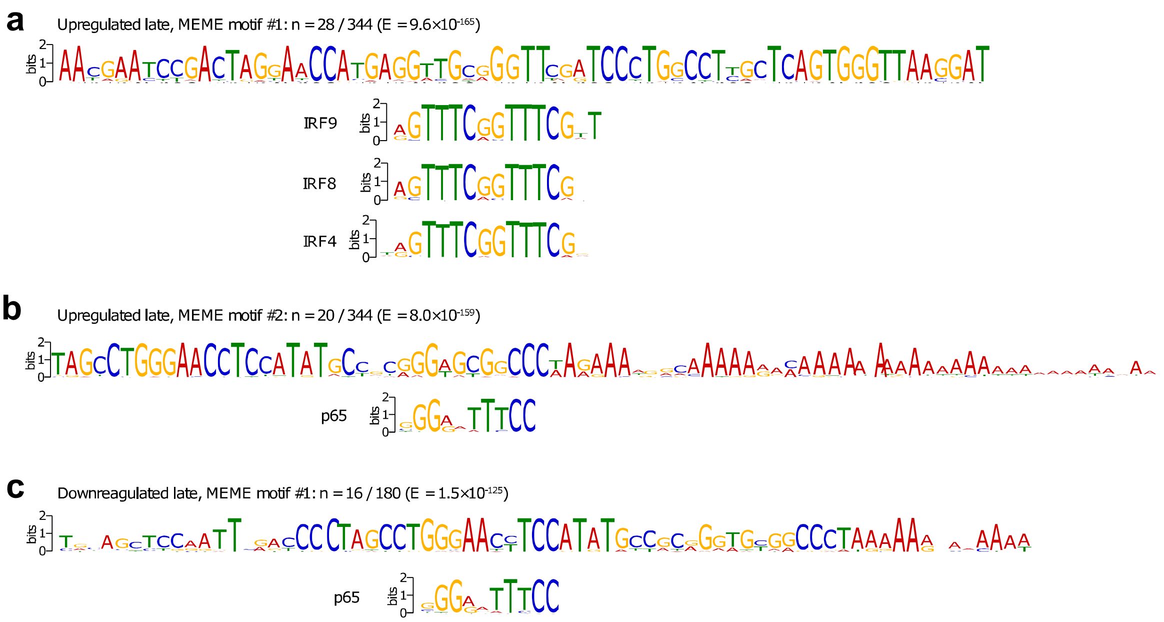

Supplementary Figure 2. Comparison of top-scored MEME motifs enriched in promoters of deregulated host genes to sequences recognised by human transcription factors: (a) Motif found in 28 upregulated gene promoters similar to sequences recognised by human interferon response factors (JASPAR accessions MA0653.1: IRF9, MA0652.1: IRF8, MA1419.1: IRF4). (b) Motif found in 20 upregulated gene promoters similar to sequences recognised by human p65/RELA protein (JASPAR accessions MA0107.1). (c) Motif found in 16 downregulated gene promoters similar to sequences recognised by human p65/RELA protein (JASPAR accessions MA0107.1).
